# Editorial Note: Research on quantitative evaluation of digital economy policy in China based on the PMC index model

**DOI:** 10.1371/journal.pone.0359603

**Published:** 2026-10-01

**Authors:** The PLOS One Editors

The *PLOS One* Editors issue this Editorial Note to inform readers that the articles cited as References 25–29 appear to be irrelevant to this article.

The corresponding author stated that the references were added in response to reviewer request, and acknowledged that they do not sufficiently match the specific statements where they were cited. They provided replacement references; however, the Editors do not consider these sufficient to fully resolve the issue.

Concerns were raised regarding the peer review process for this article. The Editors investigated and concluded that the article’s publication is supported based on expert input that was independent of the concerns.
